# Investigation of evolutionary and expressional relationships in the function of the leucine-rich repeat receptor-like protein kinase gene family (LRR-RLK) in the radish (*Raphanus sativus* L.)

**DOI:** 10.1038/s41598-019-43516-9

**Published:** 2019-05-06

**Authors:** Jinglei Wang, Tianhua Hu, Wuhong Wang, Haijiao Hu, Qingzhen Wei, Chonglai Bao

**Affiliations:** Institute of Vegetable Research, Zhejiang Academy of Agricultural Sciences, Hangzhou, 310021 China

**Keywords:** Plant evolution, Abiotic

## Abstract

The leucine-rich repeat receptor-like protein kinase (LRR-RLK) plays an important role in plant development and disease defence. Although genome-wide studies of LRR-RLKs have been performed in several species, a comprehensive analysis, including evolutionary, structural and expressional analyses and their relationships to function, has not been carried out in the radish (*Raphanus sativus* L.). In this study, we identified 292 LRR-RLK genes in the *R*. *sativus* genome and classified them into 23 subgroups. The subgroups containing genes involved in defence were more likely to evolve from tandem duplication rather than whole genome triplication (WGT), had lower expression profiles and were expressed in fewer tissues than the subgroups related to development. Gene structures and conserved domains did not differ in the defence-related or development-related subgroups, but they were distinct in each subgroup. This study sheds light on the evolutionary and expressional relationships with the functions of *R*. *sativus* LRR-RLKs and provides an integrated framework for additional investigation into these functions.

## Introduction

Cell surface receptors play important roles in perceiving and processing external and internal signals that arrive at the cell surface in both plants and animals. Receptor-like protein kinases (RLKs) are one large family of cell surface receptors^1^. A typical RLK usually consists of three distinct domains: an extracellular N-terminal domain, a transmembrane domain, and a C-terminal intracellular kinase domain (KD)^2^. KDs, which contain 250 to 300 amino acid residues that fold into a three-dimensional catalytic core, are fairly conserved^3,4^. The extracellular domains are highly divergent, and they usually consist of different protein domains^2,5^. Based on the structures of the extracellular domains, RLKs can be further classified into 17 subfamilies^6^, including the LRR-RLK family and the lectin-RLK family. The LRR-RLK family, which contains leucine-rich-repeat (LRR) domains within the extracellular domain, is the largest subgroup in the RLK superfamily^5^. Based on the structures of the KD, LRR-RLKs have been further classified into approximately 23 subgroups in *Arabidopsis thaliana*^5,7,8^.

The biological functions of LRR-RLKs have been classified into two main categories: defence against pathogens and development^9–11^. For example, the *SIF2*, *IOS1* and *FRK* genes, as members of subgroup I, are involved in defence signalling^12,13^. The subgroup XII-1 members *FLS2* and *EFR* can mediate plant resistance against bacterial pathogens^14,15^ in *A*. *thaliana* as well as in rice *Xa3/Xa26* and *Xa21*, which mediate race-specific resistance to *Xanthomonas oryzae pv*. *oryzae*^16^. In addition, *NIK*, which is contained in subgroup II^17,18^ and *PEPR2*, which is a member of subgroup XI, contribute to defence responses^19^. Alternatively, *RUL1*, which is contained in subgroup III, is involved in secondary growth^20^. Subgroup V includes the *SRF* gene, which is involved in cell wall biology, and the *SCM* gene, which is related to root hair specification^21,22^. In addition, subgroup XIIIa contains two *FEI* genes that regulate cell wall development via signalling pathways^23^, while subgroup XIIIb includes *ERECTA* and *ERECTA-LIKE* genes that are related to stomatal development and organ size regulation^24^. In addition, *CLV1*^25^, *BRI1*^26^ and *HAESA*^27^, which are distributed in subgroups II and XI, are involved in plant development. Several LRR-RLKs play roles in both development and defence because of the crosstalk between these two signalling pathways or the recognition of multiple ligands by the same receptor^28^. One example is *BAK1*, which is involved in developmental regulation via the interaction with BRI1 and is involved in defence via the interaction with FLS2^26^.

Because of the complexity of plant development as well as the responses to environmental and complementary functions between different proteins, the function of very few LRR-RLKs has been studied, especially in non-model plants^29^. Therefore, using bioinformatic tools to systematically study the evolution, expression and potential functions of the differentiation of LRR-RLKs helps to improve our understanding of their functions in efficiently regulating networks during plant growth. Several studies have investigated the membership and evolution of the LRR-RLK gene family in plant species, including the soybean^6^, two *Citrus* species^30^, four Rosaceae genomes^29^ and various species^11,31^. The radish (*Raphanus sativus* L., 2n = 2 × = 18), which is a member of the Brassiceae tribe in the plant family Brassicaceae and a relative of *Brassica rapa* and *Brassica oleracea*, has a long cultivation history in the world and is an economically important crop grown worldwide^32^. However, no systematic analysis of this gene family has been carried out in *R*. *sativus*. Thus, a comprehensive investigation of the LRR-RLK genes in the *R*. *sativus* genome is needed.

In this study, we systematically identified the *R*. *sativus* LRR-RLK genes and classified them into 23 subgroups. A detailed analysis of the genome organization, sequence phylogeny, gene structure, conserved domains, evolutionary patterns, and expression profiling was carried out. Moreover, we investigated the evolutionary and expressional relationships in the function of these subgroups. Our results provide a framework for the further functional characterization of LRR-RLK genes in *R*. *sativus*.

## Methods

### Identification and classification of LRR-RLK genes

The *A*. *thaliana* genome and annotation data were downloaded from the TAIR10 database (http://www.arabidopsis.org/)^33^. The genome resources of *R*. *sativus*^34^, *B*. *rapa*^35^ and *B*. *oleracea*^36^ were downloaded from the BRAD database (http://brassicadb.org/brad/)^37^.

To identify LRR-RLK genes, a set of proteins from the draft genome of *R*. *sativus* was scanned using hmmsearch from the HMMER v3.1 suite^38^ using a Hidden Markov Model (HMM) corresponding to the Pfam database 31.0^39^ pkinase domain model (PF00069) and pkinase_Tyr domain model (PF07714). The credible RLK proteins were generated with an E-value cutoff of 1 × 10^−5^ and coverage of the Pfam domain models of at least 50% from the raw screening proteins^7,29^.

To classify the RLKs identified into subfamilies, previously defined HMMs of different RLK subfamilies (https://github.com/lileiting/Plant_Pkinase_fam.hmm) were used to scan the RLK proteins^7,29^. The proteins were assigned to subgroups according to their best matched HMMs with an E-value cutoff of 1 × 10^−5^, which was referred to Fischer *et al*.^11^ and Shiu *et al*.^9^ The LRR-RLK genes of *A*. *thaliana*, *B*. *rapa and B*. *oleracea* were also identified using the same methods.

### Localization of LRR-RLK genes in the *R*. *sativus* genome

To construct physical maps indicating the distribution of LRR-RLK genes, genome localization details for the *R*. *sativus* LRR-RLK genes were collected from the annotation information. The MG2C (http://mg2c.iask.in/mg2c_v2.0/) was used to visualize the LRR-RLK genes on nine chromosomes^40^.

### The phylogenetic tree construction of LRR-RLKs

Multiple sequence alignment of complete amino acid sequences was performed using MUSCLE software^41^. The MEGA-X program^42^ was used to construct a Neighbour-joining (NJ) phylogenetic tree using the Jones-Taylor-Thornton (JTT) model with 500 bootstrap replicates. The uniform rates and homogeneous lineages were adopted, and the partial deletion with a site coverage cutoff of 70% was used for gaps/missing data treatment. The online tool iTol^43^ was used to clearly show the subfamily branches after the classification of all proteins.

### Conserved motifs and gene structure analysis

The motifs for LRR-RLK genes were searched using MEME software^44^. The default number of motifs to be found was set to 15, with a motif window length of 6 to 100 bp. The global perspective of the motifs in each subgroup was presented as a heatmap using the R package pheatmap^45^. The presence of transmembrane domains and signal peptides was predicted using TMHMM v2.0 (http://www.cbs.dtu.dk/services/TMHMM/)^46^ and Signalp v4.1^47^, respectively.

### Tandem duplications and syntenic analysis of LRR-RLK genes

To detect the generated mechanism of LRR-RLK genes, we analysed the tandem duplications and syntenic regions of the *R*. *sativus* and *A*. *thaliana* LRR-RLK genes. Tandem genes in *R*. *sativus* and *A*. *thaliana* were defined as those genes that (1) were separated by ten or fewer genes, (2) were within 200 kb, and (3) were orthologous genes, which were identified by the BLASTP program^48^ with an E-value cutoff < =1e-20 and coverage > =60%^8^. We identified syntenic orthologues using SynOrths software^49^, which is based on both sequence similarity and the collinearity of the flanking genes.

### Transcriptional profile analysis

For the expression profiling analysis of LRR-RLK genes, we used RNA-seq data from *R*. *sativus* tissues including flowers, siliques, leaves, stems, callus, and roots that were generated earlier and submitted to the NCBI database^32,34^. RNA-seq reads were aligned to the *R*. *sativus* genome using TopHat2^50^ software. Following the alignments, we calculated transcripts abundance on the basis of fragments per kilobase of transcript per million mapped reads (FPKM) values using Cufflinks software^51^.

## Results

### Identification, classification and distribution of LRR-RLK genes in *R*. *sativus*

**Figure 1:**
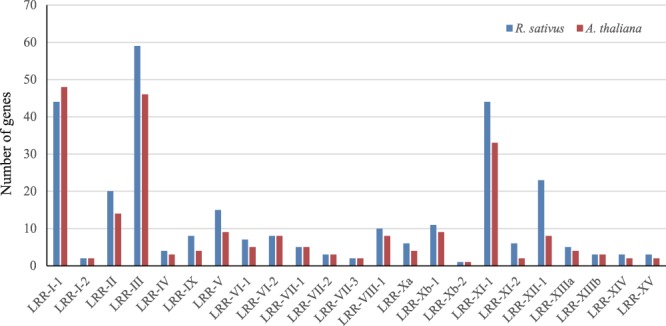
The number of LRR-RLK genes in each subgroup in *R. sativus* and *A. thaliana*.

We searched all annotated genes in the *R*. *sativus* genome for putative RLKs and identified 1540 typical RLKs in *R*. *sativus* (Supplementary Table S1). Using the HMM search approach, we classified the RLKs into 119 subfamilies, which resulted in 292 genes that were classified into the LRR-RLK family (Fig. 1 and Supplementary Table S2). We used the same method identified 225, 305 and 225 LRR-RLK genes in *A*. *thaliana*, *B*. *rapa* and *B*. *oleracea* respectively (Supplementary Table S3). The *R*. *sativus* LRR-RLK family genes were further classified into 23 subgroups, and the numbers of the subgroup genes ranged from 1 in Xb-2 to 59 in III (Supplementary Table S4). Of these subgroups, III, I-1 and XI-1 were the three largest subgroups, with 59, 44 and 44 gene numbers, respectively. The other subgroups had no more than 30 members, and Xb-2 was the smallest subgroup, with only one gene. We found that *R*. *sativus* and *A*. *thaliana* had the same subgroups; however, the ratios of the LRR-RLK gene numbers between *R*. *sativus* and *A*. *thaliana* in each subgroup ranged from 0.92 to 3. Of these subgroups, only I-1 contained fewer gene members in *R*. *sativus* than that in *A*. *thaliana*.

Compared with *B*. *rapa* and *B*. *oleracea*, most subgroups contained similar numbers of genes. While, the genes number of subgroups I-1 and XII-1 in *B*. *oleracea* were far less than that of *R*. *sativus* and *B*. *rapa*. Besides, the gene number of Xb-1 in *R*. *sativus* is less than *B*. *rapa* and *B*. *oleracea*. Although *R*. *sativus*, *B*. *rapa* and *B*. *oleracea* have same ancestral species and shared similar evolutionary history, several subgroups of LRR-RLKs have varied greatly.

**Figure 2:**
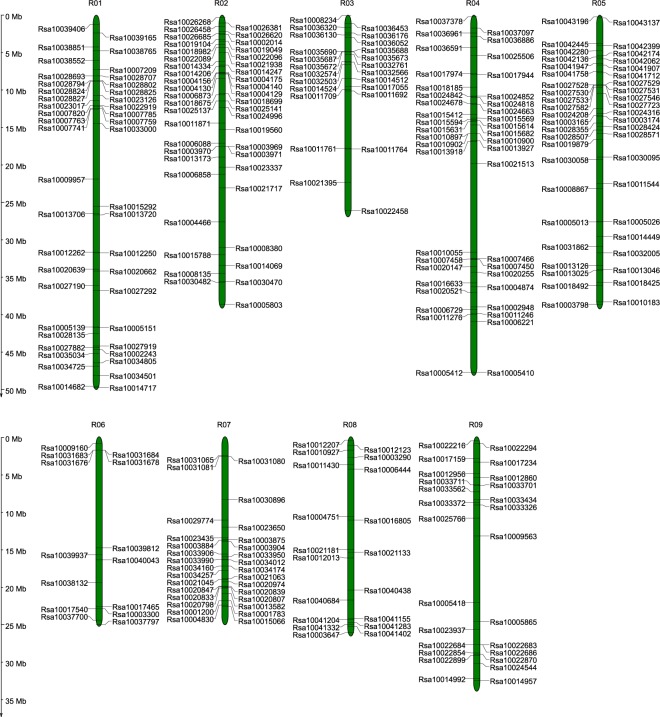
Distribution of LRR-RLK genes on R. sativus chromosomes. Green bars represent chromosomes. The black line on the olive bars indicates the location of LRR-RLK genes on chromosomes. Values corresponding to the scales on the black vertical line indicate physical distance.

We mapped 288 *R*. *sativus* LRR-RLK genes on 9 chromosomes, while the remaining 5 genes were assigned to unassembled genomic sequence scaffolds (Fig. 2). The distribution of these genes appeared to be uneven, with the distribution ratio for each chromosome ranging from 4.78% (14 genes on chromosome 6) to 15.36% (45 members on chromosomes 5). This distribution pattern is similar to that of the LRR-LRK genes in other plant species^1,6^.

### Phylogenetic analysis of LRR-RLKs

**Figure 3:**
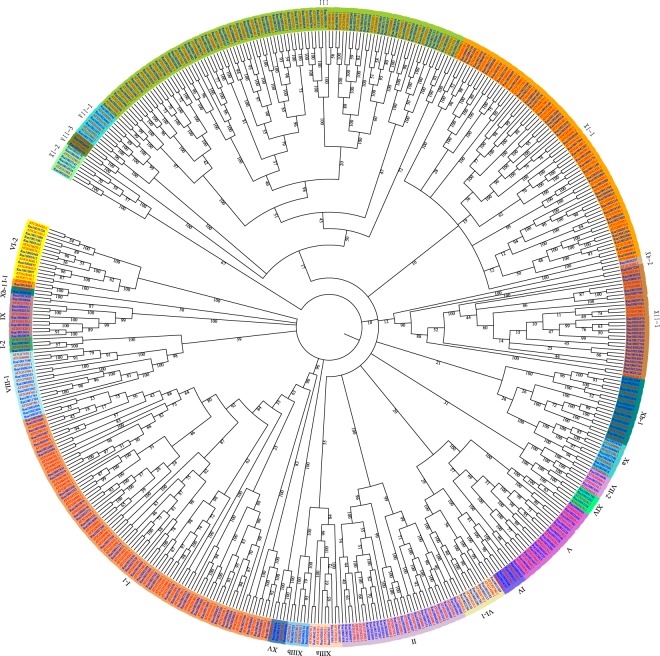
The phylogenetic tree for the LRR-RLK genes identified in *R. sativus* and *A. thaliana. R. sativus* and *A. thaliana* genes are distinguished by blue and red colours. Different subgroups are presented in different background colours.

To study the evolutionary relationships of the LRR-RLK genes in *R*. *sativus*, the LRR-RLKs from *R*. *sativus* and *A*. *thaliana* were used to construct a phylogenetic tree (Fig. 3 and Supplementary Table S5). The phylogenetic tree showed that most LRR-RLK genes in the same subfamily were classified in combination with their *A*. *thaliana* homologues. The phylogenetic tree confirmed the classification of the subgroups of *R*. *sativus* LRR-RLKs.

### Gene structure and conserved motif analysis of *R*. *sativus* LRR-RLKs

To gain insight into the structural diversity of the *R*. *sativus* LRR-RLK genes, we analysed their intron numbers (Supplementary Tables S2 and S6). The results showed that the numbers varied widely, ranging from 0 to 37. A total of 111 genes of *R*. *sativus* LRR-RLKs (37.88%) had only one intron, while 26 genes had no introns. Furthermore, a total of 86 genes had more than ten introns, and 70 genes had more than one and less than ten introns. Most of the *R*. *sativus* LRR-RLK genes in the same subgroups were conserved in terms of the intron number, while different subgroups had different patterns of intron numbers. For instance, all of the *R*. *sativus* LRR-RLK genes in subgroups III, VII, Xa and Xb contained zero, one, and two introns. In addition, all of the *R*. *sativus* LRR-RLK genes in subgroups V, VIII and XIIIb contained more than ten introns. In addition, the members of subgroups I-1, VI-2 and VIII-1 displayed a large variability in the number of introns. Most interestingly, the members of subgroup Xb-1 contained zero introns, while the members of subgroup XIIIb contained 22–25 introns. The exon/intron number indicated the conservation of gene structure within subgroups and the divergence among the different subgroups of *R*. *sativus* LRR-RLKs.

**Figure 4:**
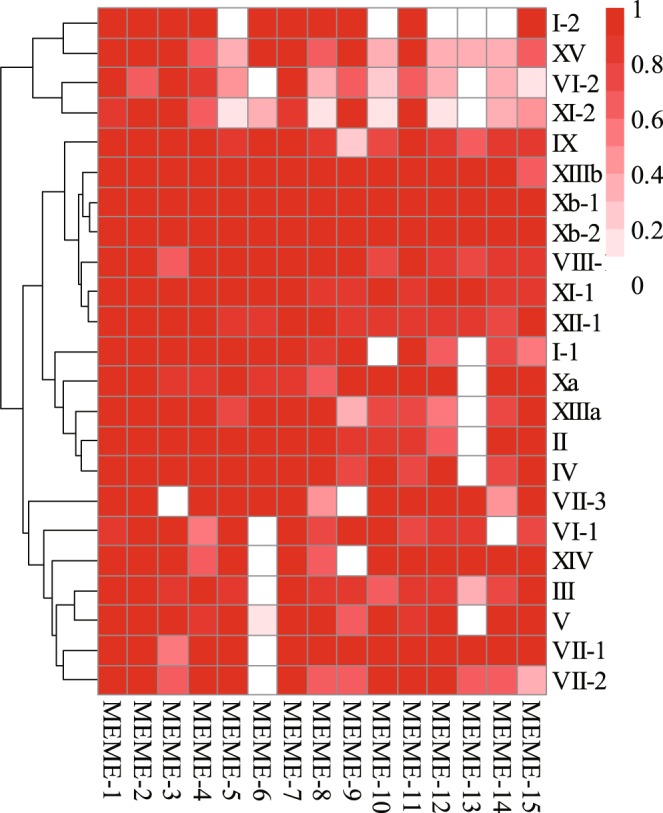
The heatmap represent the ratios of the number of genes containing the motif to the total number of genes in each subgroup, which reflects the change of motif among different subgroups as well as the conservativeness of motif within same subgroup. The darker the color, the higher the proportion.

To further understand the potential functions and diversification of the LRR-RLK genes in *R*. *sativus*, a total of 15 conserved motifs were identified and numbered 1–15 using the MEME programme^44^. The results suggested that most members in one subgroup have the same motif compositions (Fig. 4 and Supplementary Table S2). However, in terms of types and numbers, most different subgroups have different motif compositions (Fig. 4 and Supplementary Table S2). For example, the MEME-6 motif was absent in subgroups VI-1, XIV, III, V, VII-1 and VII-2, and the MEME-13 motif was absent in the other 8 subgroups. In addition, the search for the possible transmembrane (TM) domain in all the *R*. *sativus* LRR-RLKs showed that a total of 257 members had at least one TM domain, while 36 members had no TM domain, and 73 members had at least two TM domains. In addition, signal peptides were also predicted with SignalP^47^, which showed that 210 members had signal peptides (Supplementary Table S2).

### Tandem and syntenic analysis of LRR-RLK genes

Whole genome and tandem duplications provide critical sources of raw genetic material for genome complexity and evolutionary novelty^52^. In the *R*. *sativus* genome, 32 of 292 identified LRR-RLK genes, 10.92%, were demonstrated to be tandem duplications and were, distributed in 12 tandem arrays of 2–4 genes. Furthermore, 14.15% (32 out of 225) of *A*. *thaliana* LRR-RLK genes were located in tandem duplicated regions, which comprised 11 clusters in total (Supplementary Table S7). Tandem duplication clusters were distributed only among five subgroups in *R*. *sativus*. Remarkably, subgroup I-1 contained the most clusters and genes in both *A*. *thaliana* and *R*. *sativus*.

The ancestor of the diploid *Brassica* and *Raphanus* species has undergone a Brassiceae-lineage-specific whole-genome triplication (WGT) since its divergence from the *A*. *thaliana* lineage approximately 20 millions of years ago (MYA)^35,53^. The WGT was followed by diploidization that involved substantial genome reshuffling and gene losses^54,55^. We investigated the syntenic relationship of LRR-RLK genes between *A*. *thaliana* and *R*. *sativus* to trace the evolutionary history of LRR-RLK genes during the WGT. The genome-wide comparative analysis of the syntenic regions showed that 156 LRR-RLK genes in the *A*. *thaliana* genome had 225 corresponding genes in the *R*. *sativus* genome, indicating that 76.79% of the *R*. *sativus* LRR-RLK genes were retained after the WGT (Supplementary Table S8). Of the 156 *A*. *thaliana* LRR-RLK genes, 96 were shown to retain one copy, 55 retained two or three copies, and 3 genes had four copies in *R*. *sativus*. In addition, only two clusters of the 11 *A*. *thaliana* tandem duplication clusters had retained genes in *R*. *sativus*, and the others were lost during the WGT.

We also found that different subgroups had different percentages of tandem duplications and syntenic genes. For example, less than 50% of the genes in subgroups I-1, XII-1 and VIII-1 were retained during the WGT; however, in the other 12 subgroups, all the genes were retained. In addition, 40.91%, 40.00%, 33.33%, 25.00% and 17.39% of the genes in subgroups I-1, VIII-1, XI-2, VI-2 and XII-1 were present in tandem duplications, respectively (Supplementary Table S9).

### Expression profiles of LRR-RLK genes in *R*. *sativus*

**Figure 5:**
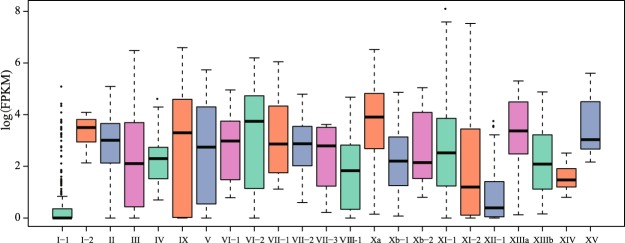
Global expression profiles in each species. Quantile boxplots (0.25, 0.75) show the distribution of the log2 transformed FPKM values of the six tissues.

To further explore the expression patterns of LRR-RLK genes in *R*. *sativus*, the transcriptomes of six different tissues (i.e., flowers, siliques, leaves, stem, callus, and roots) were collected from previous reports^32,34^ (Supplementary Table S2). In general, we found that 20.89% (61 out of 292 genes) genes were not expressed in any tissues with FPKM >1. These unexpressed genes were found in 11 subgroups (I-1, III, IX, V, VI-2, VIII-1, Xa, Xb-1, XI-1, XI-2 and XII-1) (Supplementary Fig. S1). Approximately half of the genes in several subgroups, such as I-1 and XII-1, were not expressed or were only expressed in a few tissues, and the expression profiles were very low in these subgroups (Fig. 5 and Supplementary Table S10).

## Discussion

LRR-RLKs, which are one of the largest gene families in plants, are primarily involved in plant growth and immunity in plants^56^. In this study, we identified 292 LRR-RLK genes in the genome of *R*. *sativus* and classified them into 23 subgroups. To confirm the accuracy of the identification and classification results, we used the same method to identify the LRR-RLK genes in *A*. *thaliana* as a reference. The LRR-RLK genes and their subgroups in *A*. *thaliana* identified in this study were identical with those of previous reports^7,29^, which indicates that our results were reliable. Compared with the ratio of the genome gene numbers of the two species (43,240/25,498 = 1.70)^34,57^, that of the LRR-RLK genes in *R*. *sativus* have not expanded from a global perspective, which is a result similar to that of its close species *B*. *rapa*^11^.

We analysed the retention/expansion of each subgroup and found that the ratios varied in different subgroups, which indicates that the evolutionary histories of the subgroups are different. The syntenic and tandem duplication analyses suggested that there are at least two different evolutionary mechanisms of these subgroups. One mechanism is that the LRR-RLK genes were tripled in the WGT and quickly lost in the following fractionation process, and the LRR-RLK genes were expanded via tandem duplication, such as in subgroups I-1, XII-1 and VIII-1. Another mechanism is that the LRR-RLK genes were also tripled during the WGT but were slowly lost and were retained as syntenic genes, such as in subgroups III, V and X. For subgroups I-1 and XII-1, most of the genes involved in the responses to biotic stress were currently reported in *A*. *thaliana*^11^. In addition, subgroups III and V usually contain genes involved in development^58,59^. These observations confirmed that the RLK genes involved in the stress responses were mostly duplicated by tandem duplication^9^, although few LRR-RLK genes have been functionally characterized. The recent tandem duplication generated functional redundancy in the LRR-RLK genes that can allow positive selection or relaxed negative selection of their function^9^, providing a reservoir to generate new disease resistant specificities against the fast-evolving effector genes of plant pathogens. Besides, we have found that the gene numbers of I-1, XII-1 varied greatly among the closely related species *B*. *rapa*, *B*. *oleracea* and *R*. *sativus*, which indicated that the subgroups involved in the responses to biotic stress employed different evolutionary process in different species.

Plant resistance genes need to be blocked in the absence of pathogens to avoid auto-immunity, which is detrimental to plant growth and development^60^. Notably, the genes in the defence-related subgroups, such as I-1 and XII-1, had a low expression quantity and were expressed in only a few tissues, which confirms that these subgroups are involved in the response to disease. However, no stress-treated RNA-Seq data were collected in this study. Contrasting results were observed in the developmentally related subgroups III and V, which had high expression profiles, and most of the genes were expressed in more than two tissues. Previous studies have demonstrated that introns always play an important role in cellular and developmental processes via alternate splicing or gene expression regulation^61^, and the protein domains or motifs decide the biological function of the genes. Thus, we found that each subgroup of *R*. *sativus* LRR-RLKs can differ from the others, either in the gene structure or conserved domains. However, the gene structure and conserved domains do not distinguish between defence-related or developmental-related subgroups.

Previous studies have illustrated the phylogenetic relationship, expression profiling, orthologous relationships and other characteristics of LRR-RLKs in several plants^6,29,59^, and have systematic analyzed the LRR-RLKs in plants and demonstrated that positive selection and gene expansion should be two major factors that result in differences between defence-related and development-related subgroups^10,11^. In this study, we supplemented the different evolutionary mechanisms and expressional profiles between the two groups. In total, our structural, evolutionary, and expressional analysis suggested the divergence of *R*. *sativus* subfamilies and their relationships to function. The results of this study provide insights into the evolution, expression and function of the *R*. *sativus* LRR-RLK gene family and provide a framework for the further functional investigation of these genes.

## Supplementary information

Supplementary Table

Investigation of evolutionary and expressional relationships in the function of the leucine-rich repeat receptor-like protein kinase gene family (LRR-RLK) in the radish (Raphanus sativus L.)
