# Supplementary material for: Investigation of evolutionary and expressional relationships in the function of the leucine-rich repeat receptor-like protein kinase gene family (LRR-RLK) in the radish (Raphanus sativus L.)
Source: Sci Rep. 2019 May 6;9:6937. doi: 10.1038/s41598-019-43516-9 (PMC6503142; doi:10.1038/s41598-019-43516-9)
Supplement: Supplementary file 2 — Investigation of evolutionary and expressional relationships in the function of the leucine-rich repeat receptor-like protein kinase gene family (LRR-RLK) in the radish (Raphanus sativus L.) [file 41598_2019_43516_MOESM2_ESM.docx]

**Title:** **Investigation of evolutionary and expressional relationships in the function of the leucine-rich repeat receptor-like protein kinase gene family (LRR-RLK) in the radish (*Raphanus sativus* L.)**

**Authors:** Jinglei Wang, Tianhua Hu, Wuhong Wang, Haijiao Hu, Qingzhen Wei, Chonglai Bao*

**Supplementary Information**


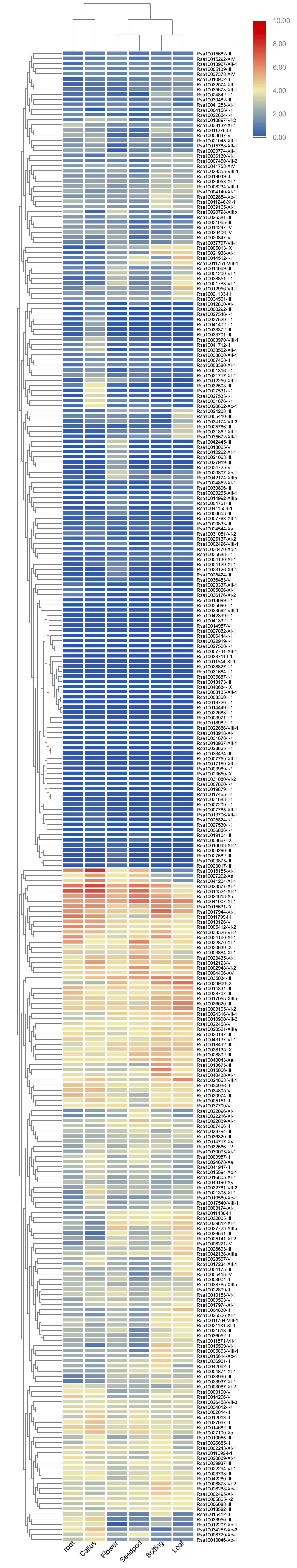


Supplementary Figure S1. The heatmap of LRR-RLKs in *R. sativus*.
